# The impact of a cardiovascular health awareness program (CHAP) on reducing blood pressure: a prospective cohort study

**DOI:** 10.1186/1471-2458-13-1230

**Published:** 2013-12-25

**Authors:** Chenglin Ye, Gary Foster, Janusz Kaczorowski, Larry W Chambers, Ricardo Angeles, Francine Marzanek-Lefebvre, Stephanie Laryea, Lehana Thabane, Lisa Dolovich

**Affiliations:** 1 Department of Clinical Epidemiology and Biostatistics, McMaster University, Hamilton, ON, Canada; 2 Biostatistics Unit, St. Joseph’s Healthcare Hamilton, Hamilton, ON, Canada; 3 Department of Family Medicine, McMaster University, McMaster Innovation Park, 175 Longwood Road South, Hamilton, ON L8P 0A1, Canada; 4 CRCHUM and Département de médecine de famille et de médecine d’urgence, Université de Montréal, Montreal, Canada; 5 Institut de recherche Élisabeth-Bruyère Research Institute, Bruyère Continuing Care and University of Ottawa, Ottawa, ON, Canada; 6 Institute for Clinical Evaluative Sciences, Toronto, ON, Canada; 7 Faculty of Health, York University, Toronto, ON, Canada; 8 Pan-American Health Organization / World Health Organization, Paramaribo, Suriname

## Abstract

**Background:**

Hypertension is an important and modifiable cardiovascular risk factor that remains under-detected and under-treated, especially in the older individuals. Community-led interventions that integrate primary health care and local resources are promising approaches to improve awareness and management of hypertension and other cardiovascular risk factors. We aimed to evaluate the effect of a community-based Cardiovascular Health Awareness Program (CHAP) on participants’ blood pressure.

**Methods:**

This study followed a cohort of community residents that participated in CHAP across 22 mid-sized Ontario communities over an 18-month period. The participants’ baseline risk factors, including blood pressure, and subsequent measures of blood pressure were recorded. We employed a bivariate linear mixed-effect model to estimate the change of systolic and diastolic blood pressure over time among the participants who attended more than two CHAP sessions.

**Results:**

Of 13,596 participants, 2498 attended more than two CHAP sessions. For those repeated participants (attending more than two sessions) initially identified with high blood pressure, the average reduction of systolic blood pressure was from 142 to 123 mmHg over an 18-month period, a monthly rate ratio of 0.992 (95% CI: 0.991,0.994; p < 0.01). Similarly, the average reduction of diastolic blood pressure was from 78 to 69 mmHg, a monthly rate ratio of 0.993 (95% CI: 0.991,0.994; p < 0.01). The average blood pressure of the participants with normal baseline blood pressure remained controlled and unchanged. We also found that older adult participants who lived alone, were diagnosed with hypertension, reported healthier eating habits, and presented with a higher baseline systolic blood pressure had significantly greater odds of attending more than one session.

**Conclusions:**

CHAP was associated with a reduction in systolic and diastolic blood pressure for those participants who attended more than one session. The magnitude of blood pressure reductions was significant clinically and statistically.

## Background

Cardiovascular disease (CVD), including heart failure, ischemic heart disease, and stroke, are the most common causes of death in Canada [1]. Treatment of CVD accounts for the largest proportion of Canadian health care expenditures [1,2]. It is estimated that the majority of CVD can be prevented through managing preventable and modifiable risk factors. One readily preventable risk factor of CVD is high blood pressure, also known as hypertension. Hypertension is among the top leading causes of death in the world [3]. In Canada, the prevalence of hypertension has remained relatively constant at around 20% of adult population over the last 3 decades (1985-2011). However, both the prevalence and incidence of hypertension increases with age. Approximately 40% of Canadians have hypertension by the age of 55 years [4-6]. For those who do not have hypertension by the age of 55 years, the residual lifetime risk of developing it is 90% [7].

Hypertension is generally asymptomatic. About 18% of adults with hypertension are unaware of their condition [8]. Another 18% of adults diagnosed with hypertension are not treated or controlled, leaving only about 64% of Canadians with adequately controlled hypertension [8]. The main barriers to optimal diagnosis, treatment and control include ‘white coat effect’ [9], ‘masked hypertension’ [10], co-morbidities [11,12], and non-adherence to both pharmacological treatments and lifestyle recommendations [13-17]. Both lifestyle and pharmacological treatments have been shown to be effective at reducing blood pressure (BP) and associated cardiovascular risk factors. Improving BP control is one of the most cost-effective health care interventions available [18]. A 10% decrease in the prevalence of hypertension would save more than $430 million per year in Canada [19]. More importantly, it would have a major impact on reducing cardiovascular morbidity and mortality.

Research suggests that community interventions that have included BP monitoring in a non-clinical setting and utilized community resources show promising results on reducing and controlling BP among hypertensive patients [19-22]. Community resources such as volunteers, pharmacists, health nurses, and local organizations are underutilized to support disease prevention and primary health care in Canada. Synergy created by connecting family physicians, pharmacists, patients, peer volunteers and local resources can lead to better health outcomes for community residents [23].

### Cardiovascular health awareness program (CHAP)

The Cardiovascular Health Awareness Program (CHAP) is a community-led approach that targets cardiovascular awareness and prevention for the older adults by providing risk assessment, repeated measurement of BP, education materials, lifestyle recommendations, and access to community resources. The program integrates primary care and local resources through the collaboration of family physicians, pharmacists, local organizations, and trained volunteer peer health educators [24]. Participation in CHAP is free and open to all residents of participating communities but individuals aged 65 years or older are explicitly targeted. Potential participants are invited by their family physicians to attend CHAP sessions held in local pharmacies. Other invitation strategies include flyers, posters, and local media campaigns through radio. The trained volunteer peer health educators, with support from a community nurse and a local program coordinator, lead CHAP sessions. In those sessions, peer health educators assist participants to measure their BP using an accurate automated device (BpTRU) and complete a CVD risk profile. Based on the risk profiles, participants may be advised to attend another session for re-assessment and follow up with their family physician [24]. The action-oriented summaries of up-to-date BP and CVD risk information are sent back to family physicians and pharmacists via an automated data system.

CHAP was developed and refined through several pilot studies and community-wide demonstrations [24-27]. It was most recently assessed in a randomized controlled trial that randomly allocated 20 mid-sized communities across Ontario, Canada to run CHAP compared with 19 control communities [22]. The result showed that CHAP communities had a significant annual reduction of 9% in cardiovascular hospital admissions in comparison with non-CHAP communities (rate ratio 0.91, 95% confidence interval 0.86 to 0.97; p = 0.002). After these promising results, CHAP continued to be implemented in 22 mid-sized communities across Ontario. The on-going goal is to develop CHAP as a sustainable community-owned program, with the aim of localizing community resources to ultimately achieve improved long-term health outcomes. CHAP is continuously evaluated by routinely collected data from the 22 communities. This is a unique data set from a real-world program that was able to follow participants for a number of months. The on-going implementation and evaluation of CHAP is an important step forward for community-led cardiovascular prevention programs which, up till now, have produced largely disappointing effects on clinical outcomes [11-13,28,29]. Today, 9 communities in Ontario have successfully adopted CHAP as their regular community program. A CHAP implementation guide targeting community end-users has been built to provide a ‘road map’ for new communities who are interested in implementing CHAP (http://www.chapprogram.ca). Part of current work on CHAP focuses on understanding the longitudinal effect of CHAP on reducing BP among hypertensive patients and the characteristics associated with participants’ participation in the program. The analyses are mainly to inform further operations of CHAP about an array of features of the program that arises in real-world implementation of the program.

In this paper, our objectives are to estimate the changes of participants’ BP over subsequent visits and examine what factors were associated with individuals’ continuous participation in CHAP.

## Methods

### Ethics statement

The participation in CHAP sessions was voluntary and did not require participants’ consent. However, blood pressure readings and other information on cardiovascular risk factors were collected and sent back to their family physician and regular pharmacist with participants’ written consent. The study and the consent procedure were approved by the research ethics boards at Bruyère Continuing Care in Ottawa, Sunnybrook Health Sciences Centre in Toronto, and McMaster University in Hamilton, Canada.

### Study design

This longitudinal cohort study followed the participants who attended CHAP sessions between May 2008 and April 2010. These CHAP sessions were delivered weekly in 22 communities after the completion of the RCT. Although those sessions were run independently of the main study, they were organized in the same way. Physician referral and local advertisements were the primary means of inviting participants. In those CHAP sessions, trained volunteer peer health educators assisted participants to take BP measurements with the BpTRU device and recorded self-reported CVD risk factors on the standardized risk profile form. An on-site community nurse was available to assess participants who had abnormal BP and trained volunteer peer health educators referred eligible participants to community pharmacists for a medication assessment. The medication assessment was a one-to-one meeting to ensure the safe and appropriate use of all types of medication. The CVD risk factors collected at baseline included the participants’ age, gender, body mass index (BMI), systolic blood pressure (SBP), diastolic blood pressure (DBP), history on transient ischemic attack (TIA), stroke, heart attack, high cholesterol and high BP, smoking status, drinking habits, eating habits (the consumption of high fat foods, vegetables, fruit and salt), stress level, physical exercises, and whether they lived alone or not. A detailed protocol of CHAP intervention can be found here [30]. The clinical outcomes of interest were SBP and DBP. Participants’ SBP and DBP were recorded during repeated CHAP sessions using standardized protocols, assistance from peer health educator volunteers, and validated automated BP devices. In keeping with the BpTRU protocol, during the first visit and subsequent visits, the BpTRU automated blood pressure measuring device independently assessed the blood pressure with volunteer peer health educators assisting with the cuff size, if required, and assisting with recording the blood pressure taken on the data collection form. The first reading was automatically discarded and the volunteer recorded the mean value of the five subsequent measurements produced by the BpTRU on the participant’s form. In CHAP, the BpTRU is set to have a one-minute interval between readings. A typical period of time required at a session was 20 minutes.

**Figure 1:**
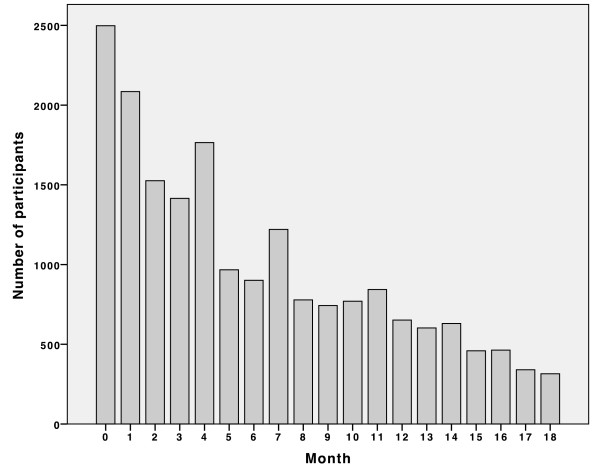
**Distribution of the number of participants over time (more than two visits).** This figure shows the distribution of the number of CHAP participants who attended more than two sessions by month. The number of participants generally decreases over time. There are more participants in some months than in previous months because CHAP being a volunteer self-directed program allows participants to attend any session.

There are two considerations for the analysis of the repeated BP measurements. First, the number of total BP measurements was generally different among participants. Second, the time interval between consecutive measurements also varied. This was due to CHAP being a volunteer self-directed program that allowed participants to attend as many sessions as they wished. The number of participants also decreased over time (Figure 1). These unequally spaced BP observations created challenges in the analysis because the time interval between consecutive measures is not the same for all participants.

### Statistical analysis

**Figure 2:**
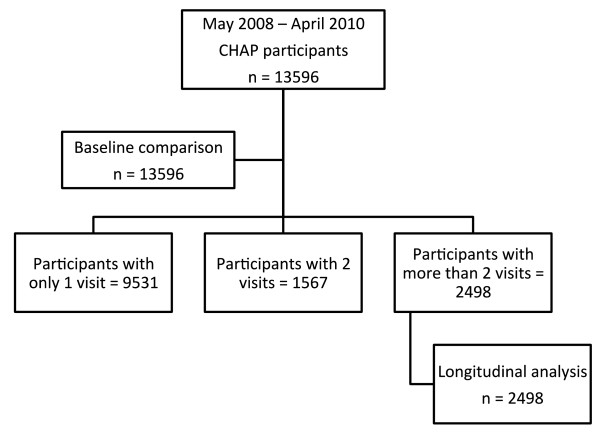
**Flowchart of the study participants.** This flowchart divides the number participants by the number of sessions: one session, two sessions or more than two sessions. It shows the number of participants for baseline comparison and the longitudinal analysis.

Participants were divided into three groups for the analysis: participants who attended only one session, two sessions, or more than two sessions (Figure 2). Mean (standard deviation) and number of count (percentage) were calculated for the continuous variables and categorical variables, respectively. The literature on longitudinal analysis suggests a minimum of at least three consecutive measures to model change through time [31]. Thus, for estimating the longitudinal effect of CHAP on reducing BP, only participants with more than two visits were included in the analysis.

A bivariate linear mixed-effect model [32-34] was used to fit SBP and DBP simultaneously by taking into account the correlation between both measures. The time of measurement was used as a continuous variable. That way, we could relate the SBP and DBP measurements to the individual time of observation to address the problem of unequally spacing between visits [35-38]. A continuous autoregressive (CAR) structure was specified for modeling the within-subject variance of the BP measurements. The CAR structure treated time between sessions as a continuous variable and allowed time-dependent correlation between measurements, e.g. the correlation between BP measurements got smaller when they were further apart. The details of the model are provided in the Additional file 1.

A logarithm transformation was applied to the outcomes to adjust for non-linear reduction over time. The SBP and DBP dropped more quickly at earlier sessions and then the reduction became smaller and smaller. In the model, we included a baseline binary variable to indicate whether the participants initially had high BP or not. According to the Canadian hypertension education program recommendations [39], the cut-off defining high SBP/DBP is ≥ 140/90 mmHg for individuals without diabetes and ≥ 130/80 for individuals with diabetes. The participants were classified in the high BP group at baseline if either SBP or DBP was above the threshold. An interaction term between the BP level indicator and the time variable was also included to allow the rate of BP change to vary between participants of different levels of initial BP.

A second analysis was conducted to examine the factors that were associated with participation in CHAP. Two logistic regression models were fitted on all participants for the odds of ever returning for another session and the odds for a new participant to be advised for the second session, respectively. The overall rate of missing observations for all variables was less than 14%. We assumed any missing observation to be missing at random (MAR) and used the multiple imputation (MI) technique to generate those missing values by baseline factors. The Monte Carlo Markov Chain (MCMC) method was used to simulate the sampling distribution for 10 imputation draws. We reported the estimate of BP change in rate ratio (RR), corresponding 95% confidence interval (CI) and p value. We chose the alpha to be 5%. All statistical analyses were performed in SAS 9.2 (SAS Institute, Cary NC).

## Results

CHAP sessions processed 13,596 participants between May 2008 and April 2010: 70% (9,531 of 13,596) attended only one session; 12% (1,567 of 13,596) attended two sessions; and 18% (2,498 of 13,596) attended more than two sessions. The baseline variables are summarized in Tables 1 and 2. The average SBP, DBP, and BMI scores on all participants were 128 mmHg, 74 mmHg, and 28 kg/m^2^, respectively and were similar across the subgroups. The average age was 67 years old and was higher among the participants who attended more than one session. Overall, 8.9% (1,101 of 12,304) participants reported a previous heart attack; 41.1% (5,048 of 12,288) reported high cholesterol; 16.0% (1,968 of 12,299) reported diabetes; and 54.0% (6,636 of 12,280) reported hypertension. The participants who attended multiple sessions reported more cardiovascular disease histories. The participants who attended more than one session reported a healthier lifestyle than those who attended only one session, e.g. less smoking, alcohol, high fat food, salt and stress, and more fruit, vegetables and physical activity. Also, the participants who lived by themselves were more likely to attend more sessions.

**Table 1:** Summary of baseline categorical risk factors

**Risk factors**		**All participants (n = 13596)**		**Participants with only 1 visit (n = 9531)**		**Participants with 2 visits (n = 1567)**		**Participants with more than 2 visits (n = 2498)**	
		**Count**	**Percentage**	**Count**	**Percentage**	**Count**	**Percentage**	**Count**	**Percentage**
Gender	Male	4987	36.7%	3525	37.2%	580	37.0%	865	34.6%
Age	≥ 65 years old	8495	62.5%	5432	57.0%	1119	71.5%	1944	77.8%
BP level	High	3975	29.3%	2744	28.8%	429	27.4%	802	32.2%
Previous TIA?	Yes	893	7.3%	581	6.7%	106	7.6%	198	8.9%
Previous stroke?	Yes	370	3.0%	239	2.8%	42	3.0%	85	3.8%
Previous heart attack?	Yes	1101	8.9%	750	8.7%	139	10.0%	208	9.4%
High cholesterol?	Yes	5048	41.1%	3433	39.8%	608	43.6%	976	44.2%
Diabetes?	Yes	1968	16.0%	1349	15.6%	216	15.5%	397	18.0%
Hypertension?	Yes	6636	54.0%	4377	50.8%	819	58.8%	1408	63.7%
Currently smoking?	Yes	1424	11.6%	1115	12.9%	132	9.5%	172	7.8%
How many times eating high fat food weekly?	3 or more	1247	10.1%	922	10.7%	124	8.9%	196	8.8%
	1-2 times	7099	57.7%	4996	57.9%	819	58.8%	1252	56.5%
	Zero	3953	32.1%	2717	31.5%	451	32.4%	768	34.7%
2+ alcoholic drinks daily?	Yes	1003	8.2%	734	8.5%	110	7.9%	157	7.1%
5+ servings of fruit and vegetable?	Yes	7482	61.2%	5126	59.8%	855	61.7%	1463	66.5%
Adding salt to food?	Often	1693	13.8%	1278	14.9%	165	11.9%	237	10.7%
	Sometimes	3059	25.0%	2140	24.9%	351	25.3%	554	25.1%
	Rarely	7476	61.1%	5160	60.2%	874	62.9%	1415	64.1%
Feeling stressed?	Often	2033	16.6%	1513	17.6%	217	15.7%	292	13.2%
	Sometimes	4553	37.2%	3210	37.4%	503	36.3%	821	37.3%
	Rarely	5645	46.2%	3865	45.0%	665	48.0%	1091	49.5%
Daily exercise?	Yes	9563	77.7%	6701	77.5%	1087	77.8%	1732	78.1%
Live alone?	Yes	3615	29.4%	2328	27.0%	456	32.6%	1398	36.8%

BP = blood pressure; TIA = transient ischemic attack.

**Table 2:** Summary of baseline continuous risk factors

**Risk factors**	**All participants (n = 13596)**		**Participants with only 1 visit (n = 9531)**		**Participants with 2 visits (n = 1567)**		**Participants with more than 2 visits (n = 2498)**	
	**Mean**	**SD**	**Mean**	**SD**	**Mean**	**SD**	**Mean**	**SD**
SBP (mmHg)	128.0	19.1	127.3	19.1	128.2	18.6	129.9	19.0
DBP (mmHg)	74.3	11.2	74.7	11.1	72.9	11.0	73.1	11.0
Age (year)	67.1	13.5	65.4	14.1	70.1	12.0	71.7	10.5
BMI (kg/m^2^)	27.9	5.4	28.0	5.4	27.7	5.4	27.7	5.3

SD = standard deviation; SBP = systolic blood pressure; DBP = diastolic blood pressure; BMI = body mass index.

**Figure 3:**
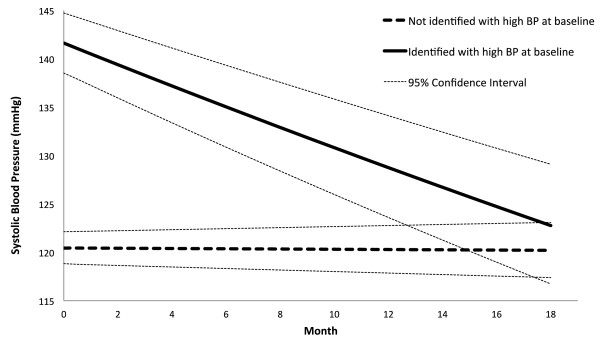
**Reduction of systolic blood pressure over time.** This figure shows the reduction of systolic blood pressure for the participants who attended more than two sessions. The reduction is presented separately for those who presented with high blood pressure at baseline (without diabetes: systolic blood pressure ≥ 140 or diastolic blood pressure ≥ 90; with diabetes: systolic blood pressure ≥ 130 or diastolic blood pressure ≥ 80) and for those who did not.

**Figure 4:**
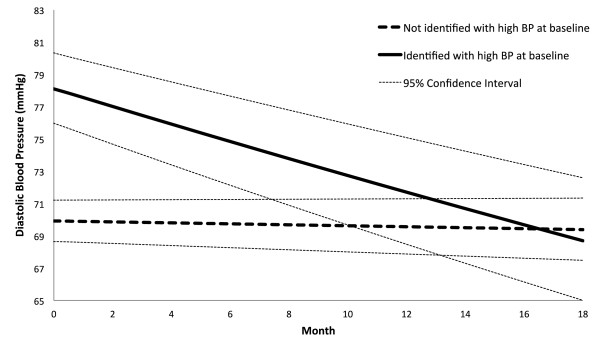
**Reduction of diastolic blood pressure over time.** This figure shows the reduction of diastolic blood pressure for the participants who attended more than two sessions. The reduction is presented separately for those who presented with high blood pressure at baseline (without diabetes: systolic blood pressure ≥ 140 or diastolic blood pressure ≥ 90; with diabetes: systolic blood pressure ≥ 130 or diastolic blood pressure ≥ 80) and for those who did not.

In Table 3, we summarize the results for estimating the BP change over repeated visits. The rate ratio (RR) estimate of BP change was interpreted as the percentage of BP change each month. For the participants initially identified with high BP, the RR of SBP and DBP was 0.9921 (95% CI: 0.9905,0.9937; p < 0.01) and 0.9929 (95% CI: 0.9914,0.9944; p < 0.01), respectively. These results showed that on average the SBP and DBP dropped 0.79% and 0.71%, respectively every month in CHAP. In contrast, the RR of SBP and DBP for the participants without high BP was 0.9999 (95% CI: 0.9993,1.0004; p = 0.69) and 0.9996 (95% CI: 0.9990,1.0001; p = 0.11), respectively. That represented an average drop of 0.01% and 0.04% on SBP and DBP, respectively every month. In summary, the average SBP/DBP for the high BP group was reduced from 142/78 mmHg to 123/69 mmHg over an 18-month period; and remained unchanged for the non-high BP group. For the participants who showed high BP at baseline, the ones who reported diabetes on average had 2.5 and 3.3 mmHg more reduction in SBP and DBP, respectively, than those who did not. In contrast, for the participants who did not show high BP at baseline, the ones who reported diabetes on average had 2.9 and 3.0 mmHg more reduction in SBP and DBP, respectively, than those who did not. Figures 3 and 4 illustrate the trend of BP reduction over time. We have examined the multi-collinearity among the baseline risk factors by calculating their variance inflation factor (VIF) and did not find any strong multi-collinearity, i.e. VIF < 10.

**Table 3:** Summary of results from the bivariate linear mixed-effect model

	**Systolic blood pressure**				**Diastolic blood pressure**			
	RR*	**95% CI**		**p value**	RR*	**95% CI**		**p value**
High blood pressure at baseline								
Baseline	141.61	(138.56	144.74)	<0.01	78.13	(75.99	80.32)	<0.01
Rate ratio	0.9921	(0.9905	0.9937)	<0.01	0.9929	(0.9914	0.9944)	<0.01
Not high blood pressure at baseline								
Baseline	120.44	(118.79	122.12)	<0.01	69.93	(68.67	71.21)	<0.01
Rate ratio	0.9999	(0.9993	1.0004)	0.69	0.9996	(0.9990	1.0001)	0.11

RR = rate ratio; CI = confidence interval.

Rate ratio was expressed as the change of current blood pressure in 1 month.

*The rate ratio was adjusted for all baseline risk factors.

The 22 mid-sized communities in this study are similar in their demographic profile, e.g. population composition, income, and residents’ education level. The distribution of BP for CHAP participants in each community is also similar but the number of total population varies. To explore the community effect on the BP change, we fitted a mixed-effect model by incorporating an additional community factor in the hierarchical structure (i.e. visit, participant and community levels). The estimated rates of BP change were similar to those obtained previously. However, the 3-level model resulted in a non-positive Hessian matrix, suggesting unstable estimates and potential over-specification of the model [40]. Thus, we report the mixed-effect model without the community factor since it provides stable estimates with a simpler covariance structure.

In Table 4, we summarize the results from the logistics regression analyses examining the factors associated with participation in CHAP. The odds of attending multiple sessions were increased by 3% (OR 1.03, 95% CI: 1.03-1.04; p < 0.01) for every year increase in age. The participants who were diagnosed with hypertension, ate more than five servings of fruit and vegetables daily, rarely added salt to food, and lived alone had 30% (OR 1.30, 95% CI: 1.19-1.42; p = 0.01), 14% (OR 1.14, 95% CI: 1.04-1.24; p = 0.01), 18% (OR 1.18, 95% CI: 1.03-1.34; p = 0.02) and 17% (OR 1.17, 95% CI: 1.06-1.28; p < 0.01) higher odds of attending more than one session than those who did not, respectively. Also, the new participants who often felt stressed were associated with 19% higher odds of being advised to attend the second session.

**Table 4:** The summary of results from the logistic regression models

	**Being advised to attend the second session**				**Attending multiple sessions**			
**Baseline factors**	**OR**	**95% CI**		**p value**	**OR**	**95% CI**		**p value**
Age	1.01	1.00	1.01	**0.04**	1.03	1.03	1.04	**<0.01**
BMI	1.00	0.99	1.02	0.44	1.00	1.00	1.01	0.78
SBP	1.02	1.01	1.02	**<0.01**	1.00	1.00	1.00	**0.02**
DBP	1.01	1.00	1.01	**0.04**	1.00	1.00	1.00	0.11
Gender								
Male^1^	1.11	1.00	1.23	0.06	1.00	0.91	1.11	0.98
Previous TIA?								
Yes^2^	0.96	0.78	1.20	0.74	0.94	0.79	1.11	0.91
Previous stroke?								
Yes^2^	1.01	0.72	1.41	0.97	1.07	0.83	1.38	0.55
Previous heart attack?								
Yes^2^	1.12	0.95	1.33	0.17	0.89	0.76	1.03	0.27
High cholesterol?								
Yes^2^	1.05	0.94	1.17	0.36	1.01	0.93	1.10	0.31
Diabetes?								
Yes^2^	1.11	0.95	1.29	0.18	0.96	0.85	1.08	0.46
Hypertension?								
Yes^2^	1.19	0.98	1.44	0.07	1.30	1.19	1.42	**0.01**
Currently smoking?								
Yes^2^	1.01	0.85	1.20	0.89	0.87	0.75	1.01	0.06
How many times eating high fat food in a week?								
Zero^3^	0.93	0.76	1.14	0.48	0.90	0.77	1.06	0.22
1-2 times^3^	0.95	0.79	1.14	0.58	0.97	0.83	1.12	0.63
Two or more alcoholic drinks a day?								
Yes^2^	0.92	0.77	1.09	0.34	0.94	0.80	1.10	0.26
At least five servings of fruits and vegetables daily?								
Yes^2^	1.10	0.98	1.22	0.10	1.14	1.04	1.24	**0.01**
Adding salt to food?								
Rarely^4^	1.06	0.92	1.23	0.43	1.18	1.03	1.34	**0.02**
Sometimes^4^	1.08	0.93	1.27	0.31	1.16	1.00	1.34	**0.05**
Feeling stressed?								
Rarely^4^	0.84	0.73	0.98	**0.02**	1.04	0.92	1.19	0.98
Sometimes^4^	0.80	0.69	0.92	**<0.01**	1.06	0.93	1.21	0.79
Daily exercise for 30 to 60 minutes most days								
Yes^2^	0.97	0.86	1.10	0.64	1.05	0.94	1.16	0.19
Live alone?								
Yes^2^	1.16	1.04	1.29	**0.01**	1.17	1.06	1.28	**<0.01**

BMI = body mass index; SBP = systolic blood pressure; DBP = diastolic blood pressure; TIA = transient ischemic attack; OR = odds ratio; CI = confidence interval.

^1^Compared with female as reference category.

^2^Compared with ‘no’ as reference category.

^3^Compared with ‘3 or more’ as reference category.

^4^Compared with ‘often’ as reference category.

Significant p values (≤ 0.05) were bolded.

## Discussion

In this study, BP of CHAP participants initially identified with high BP significantly dropped over time. An average reduction of 20/10 mmHg in SBP/DBP occurred over 18 months. Such a reduction in BP greatly lowers the risk of developing cardiovascular disease. A decrease of SBP/DBP by 10/5 mmHg reduces the risk of developing heart failure by about 50%, stroke by 38%, heart attack by 15%, and death by 10% [41]. At the same time, the participants without high BP at baseline were also well controlled in terms of their follow-up BP. These findings support our hypothesis that the use of community pharmacies and peer health educators to enhance care received at the family physician’s office overcome traditional barriers associated with measuring, treating, and controlling BP at a doctor’s office [23,24].

When compared with the participants who attended only one session, we found that the participants who attended multiple sessions generally presented with higher baseline cardiovascular risk and a healthier lifestyle. Participants who lived by themselves were more likely to come back for more CHAP sessions. From the logistic regression analysis, we found that older adult participants who lived alone, were diagnosed with hypertension previously, had healthier eating habits, and presented with higher baseline SBP had a greater likelihood of attending more than one CHAP session. In addition, for those participants who often felt stressed, they were much likely to be recommended to come back for re-assessment.

Our study was limited by a number of factors. First, risk factors were only recorded at baseline and, apart from BP, were self-reported. This prevented us from accounting for the change of risk factors over time. Second, by the pragmatic nature of the study, we cannot know which specific components of CHAP were working to reduce BP for the participants. For example, the BP reduction could be a result of an improved detection and treatment of hypertension, improved adherence to lifestyle changes or pharmacological treatments, more frequent follow-ups by family physicians initiated by CHAP for cardiovascular disease prevention, or greater efforts by integrating local resources to promote cardiovascular health within communities. Third, we showed that CHAP was effective in reducing BP in the elderly residing in mid-sized Ontario communities. These findings may not be generalizable to larger communities or urban centers where healthcare is delivered differently [22]. Fourth, CHAP was explicitly designed to target the older adults who are at least 65 years old. Thus, our findings may not hold for younger people. Fifth, the participation in CHAP was on a self-directed basis and the attrition was significant. The majority of participants attended no more than two sessions and were excluded from the longitudinal analysis of BP reduction over time. We were not able to follow those participants for their cardiovascular condition and lifestyle change at various months of the study although the baseline comparison showed that the participants with fewer visits had lower cardiovascular risk and a less healthy lifestyle. Finally, the estimates of BP reductions were derived for the population average rather than individual participants. Limited by the design, we did not protect the study from regression to the mean (RTM) bias by randomly allocating the CHAP intervention to the participants who had high BP at baseline and those who did not. However, we tried to reduce the RTM bias by recording the average BP in each measurement. We also accounted for the participants’ baseline BP when estimating the BP change. Both approaches were suggested by Barnett et al. to deal with MTR bias [42]. Using the standard formula [42], we calculated the effect of RTM in our sample to be approximately 2.0 and 0.6 mmHg for SBP and DBP, respectively. Both numbers imply a weak effect of RTM in the participants’ mean SBP and DBP.

Despite the limitations, this study has several important strengths. The CHAP intervention is evaluated by large-scale population-based data that enables longer follow-up and larger sample size. Data are rarely available for evaluation of a real-world program at this scale. The continuous and accurate monitoring of blood pressure in a familiar environment allows for a better level of diagnosis, treatment, and control of hypertension. Among the CHAP participants initially identified with elevated BP, 33% of them did not report hypertension, suggesting that these individuals might not be aware of their condition. Moreover, 11% of the participants diagnosed with hypertension previously were not receiving antihypertensive medication. When we explored the group of participants who showed high BP at baseline, we did not find any significant difference in the BP change between the ones who reported hypertension and those who did not. On average, the ones who did not report hypertension had only 0.11% (p = 0.39) and 0.17% (p = 0.15) more reduction per month in SBP and DBP, respectively, than those who did. Traditional system barriers associated with a clinical setting are overcome by the use of community pharmacies and volunteer peer health educators who are closely related to their peers. A system such as this also helps to enhance participant adherence to recommended lifestyle changes and pharmacologic treatments.

Some well-known community-wide interventions for CVD [11-13,28,29] have failed to detect significant changes in CVD risk factors or have shown only modest improvements in BP. Those studies were largely limited by small size, short duration, narrow penetration of the intervention, and a focus shifted from awareness and prevention [43,44]. In addition to overcoming those limitations, CHAP adopts a community-led, collaborative approach to primary health care and cardiovascular health awareness. It removes traditional barriers to monitoring and management of blood pressure [45]. Unlike other community BP monitoring initiatives, CHAP ensures that accurate and up-to-date information is forwarded to participants’ family physician and pharmacist [46]. The A-CHAMP in Alberta [19], a community BP program built on the CHAP model, also showed a significant BP reduction after a 6-month follow-up.

Hypertension among the modifiable risk factors of CVD represents a major public health issue and its suboptimal detection and treatment is a major burden on health care expenditures and significant CVD morbidity and mortality for those who are not adequately controlled [22]. CHAP offers a feasible, community-based, and affordable means to improve the BP management. Considering that 36% of Canadians aged 65 to 74 are uncontrolled [8], CHAP appears to present a significant step forward in the prevention of cardiovascular disease through monitoring individuals’ BP.

## Conclusions

Hypertension is highly preventable and manageable through lifestyle changes and pharmacological treatments. Our study showed that the CHAP participants initially identified with high BP experienced a significant reduction of BP during repetitive visits. Participants who presented with higher risks of developing cardiovascular diseases were more likely to attend multiple sessions. CHAP integrates community resources to promote cardiovascular health awareness and prevention among older adults to improve the diagnosis, treatment, and control of blood pressure, thus decreasing the risk of developing cardiovascular disease.

## Abbreviations

CVD: Cardiovascular disease; CHAP: Cardiovascular health awareness program; SBP: Systolic blood pressure; DBP: Diastolic blood pressure; BP: Blood pressure; CAR: Continuous autoregressive; MAR: Missing at random; MI: Multiple imputation; MCMC: Monte Carlo Markov Chain; RR: Rate ratio; CI: Confidence interval; BMI: Body mass index; RTM: Regression to the mean.

## Competing interests

The authors declare that they have no competing interests.

## Authors’ contributions

CY and LD conceived the research question; CY proposed the statistical methods, performed all analyses, interpreted the results, and drafted and revised the manuscript; JK, LWC and LD led the implementation of the CHAP study, advised on the interpretation of results and revised the manuscript; GF and LT contributed to the statistical analyses and revision of the manuscript; RA, FM and SL contributed to the implementation of the study and revision of the manuscripts; All authors have read and approved the final manuscript.

## Pre-publication history

The pre-publication history for this paper can be accessed here:

http://www.biomedcentral.com/1471-2458/13/1230/prepub

## Supplementary Material

Additional file 1 Specifics of the Bivariate Model. Click here for file
