# Supplementary material for: The impact of a cardiovascular health awareness program (CHAP) on reducing blood pressure: a prospective cohort study
Source: BMC Public Health. 2013 Dec 25;13:1230. doi: 10.1186/1471-2458-13-1230 (PMC3883556; doi:10.1186/1471-2458-13-1230)
Supplement: Additional file 1 — Specifics of the Bivariate Model. [file 1471-2458-13-1230-S1.docx]

**Additional file 1**

The bivariate linear mixed-effect model is written as,

$\left\{ \begin{aligned} {log(SBP}_{1i}\left( t \right))=\left( \beta_{10}+u_{1i} \right)+\left( \beta_{11}+b_{1i} \right)t+\beta_{12}x_{1}+\beta_{13}x_{2}+\ldots+\beta_{1\left( k-1 \right)}x_{k}+\varepsilon_{1i} \\ {log(DBP}_{2i}\left( t \right))=\left( \beta_{20}+u_{2i} \right)+\left( \beta_{21}+b_{2i} \right)t+\beta_{22}x_{1}+\beta_{23}x_{2}+\ldots+\beta_{2\left( k-1 \right)}x_{k}+\varepsilon_{2i} \end{aligned} \right.$,

where $\beta_{10}$ and $\beta_{20}$ are the common intercepts for SBP and DBP respectively; $u_{1i}$ and $u_{2i}$ are the subject-specific random intercepts; $\beta_{11}$ and $\beta_{21}$ are the fixed-effect parameters for the time variable; $b_{1i}$ and $b_{2i}$ are the subject-specific random slope for the time variable; $x^{'}s$ are the baseline characteristics treated as fixed factors; and $\varepsilon_{1i}$ and $\varepsilon_{2i}$ are the residual errors that follow a bivariate Normal distribution with a CAR covariance structure. We assume that the joint random-effect components ($u_{1i}$,$u_{2i}$,$b_{1i}$ and $b_{2i}$) between SBP and DBP follow a multivariate Normal distribution,

$\left[ \begin{matrix} \mu_{1i} \\ \mu_{2i} \\ b_{1i} \\ b_{2i} \end{matrix} \right]\sim MVN(0,D)$, where $D=\left[ \begin{matrix} \begin{matrix} \sigma_{\mu_{1}}^{2} & \sigma_{\mu_{1},\mu_{2}} & \sigma_{\mu_{1},b_{1}} & \sigma_{\mu_{1},b_{2}} \\ \sigma_{\mu_{1},\mu_{2}} & \sigma_{\mu_{2}}^{2} & \sigma_{\mu_{2},b_{1}} & \sigma_{\mu_{2},b_{2}} \\ \sigma_{\mu_{1},b_{1}} & \sigma_{\mu_{2},b_{1}} & \sigma_{b_{1}}^{2} & \sigma_{b_{1},b_{2}} \\ \sigma_{\mu_{1},b_{2}} & \sigma_{\mu_{2},b_{2}} & \sigma_{b_{1},b_{2}} & \sigma_{b_{2}}^{2} \end{matrix} \end{matrix} \right]$

where $\sigma_{\mu_{1}}^{2}$,$\sigma_{\mu_{2}}^{2}$,$\sigma_{b_{1}}^{2}$and $\sigma_{b_{2}}^{2}$are the variance of the random-effect components; and $\sigma's$ are the covariance.
